# TGF-β mediates epigenetic control of innate antiviral responses and SIV reservoir size

**DOI:** 10.21203/rs.3.rs-5626892/v1

**Published:** 2025-03-19

**Authors:** Khader Ghneim, Felipe ten-Caten, Ana Carolina Santana, Muhammad Bilal Latif, Diego Andres Diaz Dinamarca, Tamara García-Salum, Perla Mariana Del Rio Estrada, Puja Sohal, Zachary Strongin, Justin Harper, Sherrie Jean, Chelsea Wallace, Robert Balderas, Jeffrey D Lifson, Gopalan Raghunathan, Eric Rimmer, Cinthia Pastuskova, Guoxin Wu, Luca Micci, Luiz Felipe Martins Sieben, Pedro Cesar Lopes Gerum, Jessica dos Santos, Mihai G Netea, Andre van der Ven, Guido Silvestri, Daria J Hazuda, Daniel M Gorman, Bonnie J Howell, Ashish A Sharma, Mirko Paiardini, Hugo Soudeyns, Susan Pereira Ribeiro, Rafick P Sekaly

**Affiliations:** 1 Pathology Advanced Translational Research Unit (PATRU), Department of Pathology and Laboratory Medicine, Emory University School of Medicine, GA, USA.; 2 Division of Microbiology and Immunology, Emory National Primate Research Center, Emory University, Atlanta, GA, USA.; 3 Becton Dickinson, San Jose, CA, USA.; 4 AIDS and Cancer Virus Program, Frederick National Laboratory for Cancer Research, Frederick, MD, USA.; 5 Department of Discovery Biologics, Merck & Co. Inc., South San Francisco, CA, USA.; 6 Department of Pharmacokinetics, Pharmacodynamics and Drug Metabolism, Merck & Co. Inc., South San Francisco, CA, USA.; 7 Department of Quantitative Biosciences, Merck & Co. Inc., Rahway, NJ, USA.; 8 Department of Discovery Oncology, Merck & Co., Inc., Boston, Massachusetts, USA.; 9 Department of Mathematics, Cleveland State University, Cleveland, OH, USA.; 10 Department of Operations and Supply Chain Management, Cleveland State University, Cleveland, OH, USA.; 11 Department of internal medicine, Radboudumc, Nijmegen, the Netherlands.; 12 Department of Internal Medicine and Radboud Center for Infectious Diseases, Radboud University Medical Center, Nijmegen, The Netherlands.; 13 Department of Immunology and Metabolism, Life and Medical Sciences Institute, University of Bonn, Bonn, Germany.; 14 Emory Vaccine Center, Atlanta, GA, USA.; 15 Viral Immunopathology Unit, Centre de recherche Azrieli du CHU Sainte-Justine, Montreal, Canada.; 16 Department of Microbiology, Infectiology & Immunology and Department of Pediatrics, Faculty of Medicine, Université de Montréal, Montreal, Canada.; 17 Winship Cancer Institute of Emory University, Atlanta, GA, USA.

## Abstract

Immunotherapeutic approaches to eliminate latently HIV-infected cells are focused on the adaptive immune system. Herein we provide mechanistic evidence for a molecular cascade characterized by epigenetic reprogramming of innate myeloid cells and CD4 T cells. The coordinate regulation and gene expression mediated by transcription factors (TFs) IRF3, IRF7, STAT1 and C/EBPβ *versus* AP-1, promoted the development of innate antiviral immunity in these cells which was associated with control of viral load and decay of cell associated viral DNA (CA-vDNA) following analytical treatment interruption (ATI) in SIV-infected rhesus macaques (RMs) treated with anti-IL-10 and anti-PD-1. The prevalence of TGF-β/SMAD signaling in a subset of combo-treated RMs with high CA-vDNA (CA-vDNA^hi^) suppressed this antiviral activity through histone deacetylases, including HDAC11, as the latter reduced chromatin accessibility of IRFs and STATs and impeded their antiviral functions. The addition of HDAC inhibitors *in vitro* restored antiviral response in the presence of TGF-β. Induction of IL-6, a target gene of C/EBPβ, in CA-vDNA^lo^ RMs, amplified the antiviral network through IRF9, a transcription factor upstream of IRF7. We identified a similar molecular cascade in HIV elite controllers, who maintain low to undetectable viremia and small viral reservoirs without treatment. These data highlight the importance of epigenetic regulation of the host in shaping innate antiviral immune responses that control viral rebound following ATI and reduce the viral reservoir, providing insight into potential strategies for HIV cure interventions.

## Introduction

Curing HIV infection remains one of the most formidable challenges in modern medicine. Multiple approaches including “shock and kill” strategies^1^, immune interventions^2^, and cell-based therapies^3,4^ have had little impact on the reservoir or led to only transient control of viral rebound following analytical treatment interruption (ATI) of antiretroviral therapy (ART). Most of these interventions were aimed at inducing, re-shaping or restoring adaptive immunity (T and B cell responses), with little focus on rejuvenating the innate arm of the immune system known to be dysfunctional in people with HIV (PWH)^5^. Yet innate antiviral immune responses, specifically the interferon (IFN) pathway, play a critical role in the control of viral infections^6^ as they trigger the expression of IFN-stimulated genes (ISGs) that target viruses at multiple stages of their life cycle and are associated with control of HIV acquisition^7^. Of note, the timing and persistence of the IFN-induced responses is critical. The infusion of IFN-a2a in rhesus macaques (RMs) before SIV infection leads to resistance to SIV acquisition^8^ by upregulating ISGs with antiviral functions. However, during chronic viral infections when other mediators of inflammation are present (i.e. TNF-α; NF-κβ, AP-1 and their target genes), the upregulation of IFNs and ISGs can lead to deleterious outcomes in HIV and SIV infections^9,10,11^.

As part of the natural homeostasis of the immune system, cytokines with anti-inflammatory properties, such as TGF-β, play an important role in modulating immune responses. However, by dampening effector responses, these cytokines can promote the chronicity of infections. TGF-β is a pleiotropic cytokine produced by several cells of the immune system, including natural and induced Tregs. It controls T cell homeostasis and is critical to reduce the inflammation observed in acute and chronic viral infections^12^. Circulating TGF-β levels are increased in PWH^13^. Our group and others have shown the critical role of TGF-β in the establishment and maintenance of HIV persistence^14,15^, although the mechanisms downstream of TGF-β in modulating antiviral defenses are yet to be defined.

HIV elite controllers (ECs) are a rare group of PWH (0.1–2.5%) who maintain undetectable HIV viral load (VL; <50 copies/mL) for over 2 years in the absence of ART^16^. Host factors such as specific HLA subtypes, higher levels of expression of p21 mRNA in T cells, and lower levels of expression of CCR5^17–19^ explain the viremia control in the majority, but not all HIV ECs. These individuals harbor replication-competent HIV strains, thus the identification of additional immune effector mechanisms associated with antiviral immunity may provide a better understanding of VL control in HIV ECs that do not harbor genetic protective factors.

We have previously shown that the dual blockade of IL-10 and PD-1 signaling (combo-treatment) led to the control of viremia post-ATI in 9/10 treated RMs. However, only 4/10 treated RMs presented with a significantly smaller viral reservoir, which is one of the main endpoints of HIV cure strategies. Herein, we used cellular, molecular and systems immunology approaches to investigate the pathways associated with lower levels of cell-associated viral DNA (CA-vDNA^lo^) in these RMs. We found high levels of expression of interferon regulatory factors (IRFs) and C/EBPβ and their target genes in CA-vDNA^lo^ RMs. Conversely, high levels of expression of SMAD2/3 and AP-1 and their target genes and enzymes involved in epigenetic reprogramming, including HDACs, were observed in CA-vDNA^hi^ RMs. Gene signatures associated with combo treatment, innate control of viral replication and smaller reservoir size were also found in HIV ECs, suggesting the potential generalizability and the clinical translatability of this intervention to reduce the viral reservoir in people with HIV.

## Results

We previously demonstrated that infusion of a combination of monoclonal antibodies that block IL-10 and PD-1-signaling (combo treatment) in ART-treated RMs (**Extended Data Fig. 1A** - top panel) led to i) post-ATI control of VL rebound in 90% of the RMs to <1,000 copies/mL over 6 months; and ii) declining levels of cell associated viral DNA (CA-vDNA, <100 cells/10^6^ CD4^+^ T cells) in 40% of RMs^20^ over the same period (herein termed CA-vDNA^lo^ RMs) (**Extended Data Fig. 1A** – bottom panel and **Extended Data Fig. 1B**, respectively). RMs in the combo treated group could not be distinguished by the frequencies of cells with CA-vDNA pre-ATI. Moreover, both CA-vDNA^lo^ and CA-vDNA^hi^ RMs showed the same levels of plasma VL post-ATI and during the period of follow-up. We used integrated systems immunology approaches to identify the mechanistic underpinnings that led to viral control and a decay in CA-vDNA levels in combo-treated RMs. We hypothesized that a poised immune antiviral state in myeloid and CD4^+^ T cells pre-ATI prevented the *de novo* seeding of bystander cells post-ATI by rebounding virus, resulting in the CA-vDNA^lo^ phenotype observed in a subset of these RMs.

### Antiviral gene signatures are induced by combo treatment and are associated with virologic control.

**Fig 1.**
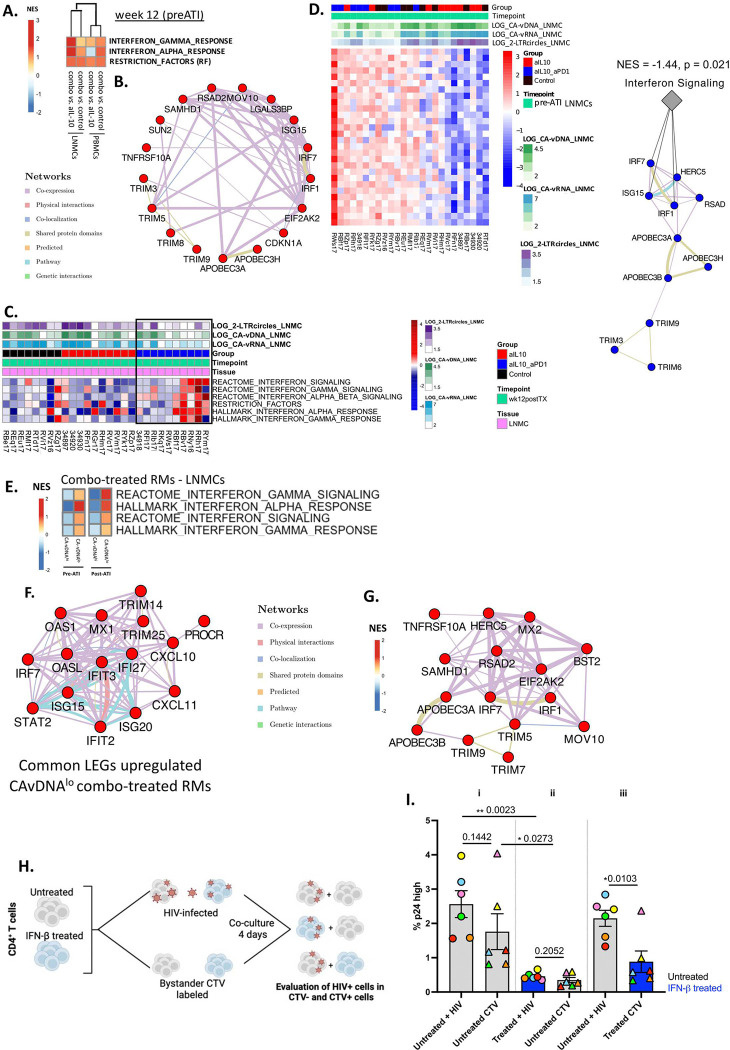
IFN signaling is the major pathway induced in PBMCs and LNMCs of combo-treated RMs pre-ATI and is inversely correlated with virological readouts 24 weeks post-ATI. **A.** GSEA was used to identify pathways (MSigDB, Hallmark) that significantly (p ≤ 0.05) differentiated combo-treated RMs from control RMs or RMs treated only with aIL-10 pre-ATI in PBMCs and LNMCs. Heatmap illustrates NES ranging from high (red) to low (blue). Rows represent gene-sets and columns represent contrasts observed in PBMCs and LNMCs for pathways of the IFN module. **B.** Network inference (Cytoscape, Genemania) was used to plot the common IFN significant LEGs (p < 0.05) of pathways in Fig 1A that were up-regulated in PBMCs and LNMCs of combo-treated RMs (red circular nodes). Connecting lines highlight the type of gene-gene interactions, color-coded by network type. **C.** Heatmap illustrates SLEA scores for significant (p < 0.05) Reactome/Hallmark IFN signaling pathways and RFs pre-ATI in LNMCs from control (black), aIL-10 only (red) and combo-treated RMs (blue). Rows represent pathways and columns represent individual samples. A red-white-blue gradient was used to depict the relative SLEA scores of the pathways, where blue represents low and red represents high relative levels of expression. The levels of CA-vRNA, CA-vDNA and 2-LTR circles (white to blue, green, purple, respectively) 24 weeks post-ATI were plotted as annotations at the top of the heatmap. Note the higher enrichment of IFN pathways in a subset of combo-treated RMs with low SIV CA-vDNA levels (boxed). **D.** Left panel: heatmap illustrating results of regression analysis of gene expression (top 50 genes) in LNMCs as a function of viral outcomes, *i.e.* log SIV CA-vDNA (green), log CA-vRNA (blue), and log 2-LTR circles (purple) 24 weeks post-ATI (p < 0.05). Rows represent genes and columns represent individual RMs: controls (black), aIL-10 only (red), and combo-treated (blue). Gene expression levels were centered on zero and ranged to a standard deviation of 1 (z-score). A blue-white-red gradient was used to depict relative gene expression, where blue represents low expression and red high expression. Right panel: network representing the Gene Ontology functional annotation of the top IFN-stimulated genes with antiviral capacity that were negatively correlated (NES = −1.44, p = 0.021) to outcomes at week 24 post-ATI. Blue nodes represent inverse correlation with virological outcomes. **E.** IFN and antiviral pathways (p ≤ 0.05) persistently upregulated in CA-vDNA^lo^ RMs pre- and postATI. Heatmap illustrates NES for IFN related pathways. Rows represent gene sets and columns represent comparisons at both pre and post ATI timepoints in LMNCs. **F.** LEGs up-regulated at weeks 0 and 24 in CA-vDNA^lo^ RMs are represented in the network. **G.** Network inference (Cytoscape, Genemania) was used to plot the common IFN-associated genes that were up-regulated in PBMCs and LNMCs (significant LEGs). Red circular nodes represent up-regulated genes in combo-treated RMs. Lines highlight gene-gene interactions. **H.** Experimental design. Cells were pre-treated or not with IFN-β, then divided into 2 pools. Unlabeled CD4^+^ T cells were infected with HIV by spinoculation. Uninfected cells were labeled with CTV. On day 2, unlabeled HIV-infected cells and CTV-labeled HIV-uninfected cells were mixed (1:1 ratio) and cocultured for 4 days in the presence of saquinavir. **I.** Frequencies of cells positive for HIV p24 were measured by flow cytometry on day 6 in CTV^−^ and CTV^+^ cells. Gray bars: untreated conditions; blue bars: IFN-β-treated conditions. Circles: HIV-infected CD4^+^ T cells; triangles: CTV-labeled bystander CD4^+^ T cells. *p < 0.05, **p < 0.005, paired t-test. 2-LTR circles: 2 long terminal repeat circles; ATI: analytical treatment interruption; CA-vDNA: cell associated viral DNA; CA-vRNA: cell associated viral RNA; CTV: cell trace violet.; IFN: interferon; GSEA: gene set enrichment analysis; LEGs: leading-edge genes; LNMCs: lymph node mononuclear cells; NES: normalized enrichment score; PBMCs: peripheral blood mononuclear cells; RFs: HIV restriction factors; RMs: Rhesus macaques; SIV: simian immunodeficiency virus; SLEA: sample level enrichment analysis

Unsupervised module analysis (**Extended Data Fig. 1C**) and gene set enrichment analysis (GSEA) (Fig. 1A) revealed that IFN signaling (Hallmark & Reactome, MSigDB) was the module of genes that was uniquely upregulated in lymph node mononuclear cells (LNMCs) and peripheral blood mononuclear cells (PBMCs) from combo-treated RMs pre-ATI when compared to the 2 other groups, (*i.e.*, RMs treated with aIL-10 alone and ART-only controls) (**Supplementary Table 1, 2**). The network of differentially expressed genes included known viral restriction factors (RFs) (APOBECs, TRIMs, ISG15 - Fig. 1B), all of which are targets of the TFs IRF7 and STAT1^21^. The inferred transcriptional activity of IRF7 and STAT1 in combo-treated RMs also included heightened expression of genes that regulate cell proliferation, DNA damage and cell cycle arrest (PSMA5/6, PSMB8/10, RAD1, BARD1) (**Extended Data Fig. 1D–E**). These gene signatures are inversely associated with levels of CA-vDNA (Fig. 1C).

Of note, myeloid and T cells from CA-vDNA^lo^ RMs presented significantly higher frequency or per cell expression level of pIRF3/7 (**Extended Data Fig. 1F**), which was mainly observed in LNMCs. Regression analysis (gene expression ~ outcomes) in LNMCs revealed that expression of several antiviral ISGs (IRF7, APOBECs, TRIMs) pre-ATI was inversely correlated with levels of CA-vDNA, CA-vRNA, and 2-LTR circles 24 weeks post-ATI (Fig. 1D, **Supplementary Table 3**), supporting the role of IFNs in defining virologic outcomes. In fact, the active phosphorylated form of IRF3 (pIRF3) in classical monocytes was inversely correlated with VL 24 weeks post-ATI, and pIRF7 in central memory CD4^+^ T cells (T_CM_) pre-ATI, was inversely correlated with viral load (VL), and levels of CA-vDNA and CA-vRNA 24 weeks post-ATI (**Extended Data Fig. 1G–H**). Integration of flow cytometry and bulk RNA-Seq data pre-ATI (**Extended Data Fig. 1I** – left panel) provided a significantly more accurate prediction of lower CA-vRNA levels post-ATI in the combo-treated RMs as compared to the other treated groups than each individual dataset alone (from p = 0.021 to p = 0.0093; **Extended Data Fig. 1I** - right panel). Altogether, we showed that the upregulation of IFN signaling, of genes with antiviral activity, and of RFs, all downstream of IFNs, were observed exclusively in innate and adaptive immune cells from LNMCs and PBMCs from combo-treated RMs belonging to the CA-vDNA^lo^ subgroup.

### Antiviral gene signatures in CA-vDNA^lo^ RMs persisted at least 6 months post-ATI.

We next monitored the durability of the pre-ATI, IFN-related pathways and antiviral gene signatures in PBMCs and LNMCs that were associated with the CA-vDNA^lo^ phenotype. In LNMCs, increased levels of IRF3 and STAT1 phosphorylation were observed in T naïve and CD4^+^ and CD8^+^ T_CM_ from combo-treated RMs 24 weeks post-ATI (**Extended Data Fig. 2A**). Importantly, several IFN-related pathways, including Hallmark IFN alpha/gamma signaling and Reactome IFN signaling, which were up-regulated in CA-vDNA^lo^ RMs pre-ATI, were maintained 24 weeks post-ATI (Fig. 1E, **Supplementary Table 4**) as compared to CA-vDNA^hi^ RMs. IRF7, its target genes, host antiviral RFs ISG15/20, OAS1/L, MX1, and chemokines CXCL10/11 were commonly upregulated in CA-vDNA^lo^ RMs pre- and post-ATI (Fig. 1F). Taken together, these data reveal that combo treatment led to early induction (pre-ATI) of IFN-related pathways and antiviral genes which were inversely correlated with SIV readouts, and that these signatures were persistent up to 24 weeks post-ATI in CA-vDNA^lo^ RMs. On the other hand, these signatures *i.e.*, IFN signaling, were significantly reduced in CA-vDNA^hi^ RMs at both time points analyzed (pre-ATI and 24 weeks post-ATI).

### Administration of aPD-1 is required for the induction of antiviral signatures.

Although we did not include a solo aPD-1 arm in this study, previous reports showed a transient impact of this intervention on VL control^22–27^. In our study, RMs treated only with aIL-10 also did not show VL control post-ATI^28^(**Extended Data Fig. 1A** – bottom panel), suggesting that it was the synergistic combination regimen of aPD-1 and aIL-10 that led to the control of VL and decay of CA-vDNA levels. To identify the molecular pathways triggered by the combined effect of aPD-1 and aIL-10 as compared to aIL-10 alone, differential gene expression analysis of bulk RNA-Seq in LNMCs and PBMCs pre-ATI was performed. A total of 2361 (626 upregulated) and 1488 (938 upregulated) differentially expressed genes (DEGs) were significantly modulated in LNMCs and PBMCs from combo-treated RMs, respectively, as compared to RMs treated only with aIL-10 (**Extended Data Fig. 2B, Supplementary Table 1**). Functional pathway analysis of the DEGs showed that RFs and IFN-induced pathways (IFN-α and IFN-γ), all with antiviral effector functions, were enriched pre-ATI in LNMCs and PBMCs from combo-treated RMs (Fig. 1A), and included DEGs such as APOBECs, TRIMs, and MX2 (Fig. 1G). This suggested that the transcriptional upregulation of IFN pathways in combo-treated RMs was triggered by the addition of aPD-1 to aIL-10. Upregulation of these genes in LNMCs from combo-treated RMs distinguished between RMs with low and high levels of CA-vDNA, CA-vRNA, and 2-LTR circles 24 weeks post-ATI (**Extended Data Fig. 2C**). These results showed that the addition of aPD-1 to aIL-10 led to the upregulation of the IFN pathway and antiviral genes that limited the capacity of the virus to disseminate to other cells post-ATI, resulting in lower levels of CA-vDNA in lymphoid tissues.

To experimentally confirm the role of IFNs in protecting bystander CD4^+^ T cells from infection, CD4^+^ T cells isolated from PBMCs of healthy human volunteers were left unstimulated (untreated) or were pre-treated with IFN-β (treated). Each culture was subdivided into two pools: cells in the first pool were infected with HIV, while those in the second pool were labeled with Cell Trace violet (CTV) and were used as bystander target cells. These pools were co-cultured as shown (Fig. 1H). Pre-treatment with IFN-β prior to co-culture led to the upregulation of ISGs (*e.g.*, IFIT1, APOBEC3G, MX2) as compared to untreated conditions (**Extended Data Fig. 3A**). After 4 days of co-culture, i) untreated CD4^+^ T cells and untreated bystander (CTV-labeled) CD4^+^ T cells were both productively infected with HIV (2.5–3% p24^+^ cells); ii) treated CD4^+^ T cells were protected from infection, presenting with significantly lower frequencies of HIV-p24^+^ cells than untreated CD4^+^ T cells (i). This led to significantly lower levels of infection of untreated CTV-labeled bystander CD4^+^ T cells (~0.5% p24^+^ cells); and iii) treated CTV-labeled bystander CD4^+^ T cells were protected from infection in the presence of untreated, productively infected CD4^+^ T cells (2–2.5% HIV^+^ cells) (Fig. 1I). Collectively, these outcomes are similar to CA-vDNA^hi^ RMs, who failed to express antiviral signatures after treatment, resulting in elevated viral DNA. Conversely, the induction of antiviral signatures pre-ATI in CA-vDNA^lo^ RMs promoted refractoriness to infection, sparing bystander target cells from infection post-ATI when viremia was below 1,000 copies/mL.

### TGF-β plasma levels and gene signatures are associated with a network of epigenetic enzymes that impact the IFN response.

**Fig 2.**
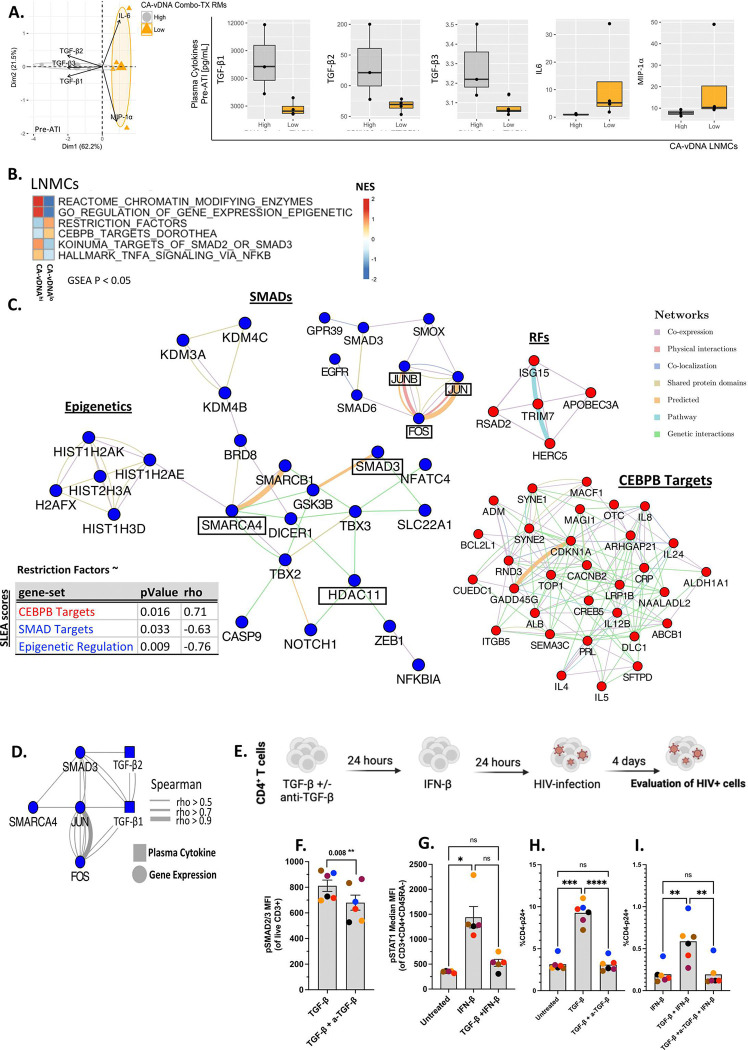
Transcriptomic analysis of PBMCs and LNMCs of combo-treated RMs with lower CA-vDNA levels reveals higher IFN-associated restriction factor signaling and lower SMAD signaling and epigenetic signatures. **A.** Quantification of TGF-β isoforms in plasma from combo-treated RMs pre-ATI who became CA-vDNA^lo^ or CA-vDNA^hi^ 24 weeks post-ATI. Left panel: PCA highlighting significantly different (Wilcoxon test, p < 0.05) levels of plasma cytokines pre-ATI between CA-vDNA^hi^ and CA-vDNA^lo^ RMs (24 weeks post-ATI). Right panel: Jitter plots highlighting the different levels of all TGF-β isoforms (*i.e.*, TGF-β1, TGF-β2, TGF-β3), IL-6 and MIP-1α pre-ATI in CA-vDNA^hi^ and CA-vDNA^lo^ RMs 24 weeks post-ATI. **B.** GSEA was used to test the enrichment of pathways (MSigDB c2, c7, Hallmark) that distinguished CA-vDNA^lo^ from CA-vDNA^hi^ RMs. The heatmap illustrates NES of pathways related to epigenetic modifications, SMAD2/3 targets, innate antiviral signatures, and inflammation (p < 0.05). The NES scale ranges from low (blue) to high (red). Columns represent relative enrichment in CA-vDNA^hi^ (left) and CA-vDNA^lo^ RMs (right) whereas rows represent pathways. **C.** Network inference (Cytoscape, Genemania) was used to plot the significant LEGs of SMAD targets and epigenetic signatures (both downregulated, blue nodes), and RFs and C/EBPβ target genes (both upregulated, red nodes) pre-ATI in LNMCs from CA-vDNA^hi^ *versus* CA-vDNA^lo^, respectively. Edges highlight gene-gene interactions. The table highlights the Spearman correlation coefficients and p values between the RF pathways and the other pathways represented in the gene network. **D.** An integrated model was generated in the R Mixomics package (http://mixomics.org/)^60^ using plasma cytokine and bulk RNA-Seq data. This integrated network highlights the Spearman correlation between gene expression (blue circular nodes) and plasma cytokines (blue square nodes). The thickness of connecting lines represents the Spearman correlation coefficient associated with marker pairs. **E.** Experimental design. CD4^+^ T cells were pre-treated with TGF-β, with or without an anti-TGF-β antibody for 24h, followed by stimulation with IFN-β (24h). CD4^+^ T cells were then infected with HIV by spinoculation. HIV p24 levels were evaluated by flow cytometry 4 days post-infection. **F.** Bar graph depicting MFI of phosphorylated SMAD2/3 in live CD3^+^ cells from healthy human donors (n = 6) following stimulation with TGF-β and stimulation with TGF-β in presence of anti-TGF-β antibody. **G.** Bar graph depicting MFI of phosphorylated STAT1 in CD3^+^CD4^+^CD45RA^−^ cells from healthy human donors (n = 6) under unstimulated conditions, stimulation with IFN-β, and pre-stimulation with TGF-β followed by stimulation with IFN-β. **H.** Bar graph depicting frequencies of p24^+^ cells in cultures of CD3^+^CD4^lo^ cells under different stimulation conditions (UNS vs TGF-β vs TGF-β + anti-TGF-β antibody). **I.** Bar graph depicting frequencies of p24^+^ cells in cultures of CD3^+^CD4^lo^ cells under different stimulation conditions (IFN-β vs TGF-β + IFN-β vs TGF-β + IFN-β + anti-TGF-β antibody). *p < 0.05, **p < 0.005, ***p < 0.0005, ****p < 0.0001, paired t-test. CA-vDNA: cell associated viral DNA; FMO: fluorescence minus one; GSEA: gene set enrichment analysis; IFN: interferon; LEGs: leading-edge genes; MFI: median fluorescence intensity; NES: normalized enrichment score; PCA: principal component analysis; RFs: restriction factors; RMs: Rhesus macaques; UNS: unstimulated.

Among the 28 cytokines quantified in plasma, CA-vDNA^hi^ RMs presented significantly higher levels of all TGF-β isoforms (*i.e.*, TGF-β1, TGF-β2, and TGF-β3) pre-ATI as compared to CA-vDNA^lo^ RMs. TGF-β inhibits the production of type-I and type-II IFNs^29^. In addition, CA-vDNA^lo^ RMs presented significantly higher plasma levels of MIP-1α, an inhibitor of HIV entry^30^, and IL-6, which triggers IFNs^31,32^ (Fig. 2A, **Supplementary Table 5**). Supporting these findings, GSEA revealed that pathways related to TGF-β signaling, such as SMAD2/3 targets (AP-1: Fos/Jun), and/or to inflammation (TNF signaling, *i.e.*, NFKBIA, TNFSF9, and IL1A) were significantly upregulated in CA-VDNA^hi^ RMs (Fig. 2B, **Supplementary Table 6**). Of note, SMAD2/3 and AP-1 translocate to the nucleus to drive the expression of TGF-β^19^. Bulk RNA-Seq on LNMCs pre-ATI also revealed that pathways that map to the enzymatic machinery that regulates chromatin accessibility and structure, (Reactome Chromatin Modifying Enzymes, GO Regulation of Gene Expression Epigenetic) were upregulated in CA-vDNA^hi^ RMs (Fig. 2B–D). Lysine demethylases (KDM3A, KDM4B), the SWI/SNF complex (SMARCA4/BRG1, SMARCB1), and HDAC11 are all inhibitors of both type I and type II IFN transcription^33^. Expression of these effectors of epigenetic reprogramming (SMARCA4 and HDAC11) was associated with plasma levels of TGF-β and TGF-β signaling (SMAD3) (Fig. 2D). In contrast, the Restriction Factor pathway, which includes >50 genes with antiviral activity such as ISG15, APOBEC3A, and TRIM7 and the C/EBPβ pathway, which controls the expression of IL-6 and inhibits the expression of AP-1, were specifically upregulated in CA-vDNA^lo^ RMs (Fig. 2B–C). Of note, the normalized enrichment scores (NES) of TGF-β signaling (through SMAD2/SMAD3) and pathways that include enzymes involved in chromatin remodeling were significantly and negatively correlated with the IFN-induced antiviral pathways (p = 0.033, rho = − 0.63)^34^. Conversely, the expression of RFs and C/EBPβ pathways were positively correlated with one another (p = 0.016, rho = 0.71). These data point to TGF-β and TGF-β signaling as upstream modulators of chromatin remodeling and antiviral responses resulting in the suppression of the innate antiviral response that prevents SIV dissemination.

### *Ex vivo* and *in vitro* validation of the interplay between TGF-β, HDACs and IFN antiviral activity.

To demonstrate the capacity of TGF-β to dampen the antiviral activity of IFN signaling, we isolated memory CD4^+^ T cells from healthy human volunteers and cultured them under various conditions (untreated; TGF-β only; IFN-β only; pre-stimulated with TGF-β followed by IFN-β with or without anti-TGF-β antibody) (Fig. 2E). Treatment *in vitro* with anti-TGF-β significantly decreased TGF-β-induced phosphorylation of SMAD2/3 (pSMAD2/3 - Fig. 2F). Pre-stimulation of CD4^+^ T cells with TGF-β for 24 hours impeded the upregulation of pSTAT1 by IFN-β (Fig 2G). CD4^+^ T cell co-cultures (all the aforementioned conditions) were infected *in vitro* with HIV. After 4 days in culture, expression of HIV Gag p24 was evaluated by flow cytometry. Cultures treated only with TGF-β displayed significantly higher frequencies of p24^+^ cells as compared to untreated cultures or cells cultured in the presence of anti-TGF-β (Fig 2H). Cultures stimulated with TGF-β prior to IFN-β treatment exhibited significantly higher frequencies of p24^+^ cells compared to cells treated with IFN-β alone or cultures containing anti-TGF-β (Fig. 2I). These data provide experimental validation for the specific role of TGF-β in dampening antiviral signatures and promoting HIV infection. These findings confirms that TGF-β is a potent antagonist of IFN-induced antiviral responses^29^ providing a mechanistic framework for our *in vivo* observations in CA-vDNA^hi^ RMs.

**Fig 3.**
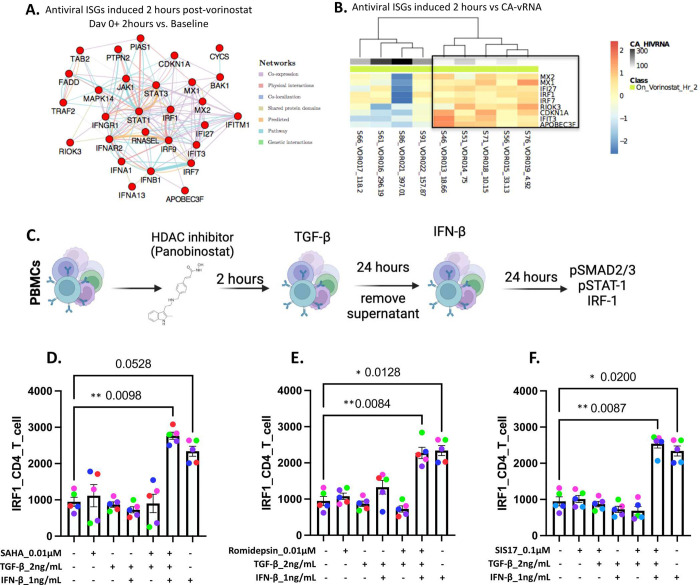
HDAC inhibition leads to enhanced interferon signaling. **A.** Co-expression network (cytoscape) illustrates a set of ISGs up-regulated 2h post the first dose of vorinostat (day 0 + 2h) and 2h after the seventh dose of vorinostat (day 7 + 2h) compared to baseline (no administration). Red circular nodes indicate significantly up-regulated genes (LIMMA, p < 0.05). Colored edges represent gene-gene interaction. **B.** Heatmap illustrates results of regression analysis between gene expression measured 2h post-vorinostat administration and viral outcome cell associated HIV RNA (white to black gradient) measured on day 84 (70 days after discontinuation of vorinostat). Rows represent genes (p <0.05) and columns represent individual samples. Gene expression levels were centered at zero (z-score). A blue-white-red gradient was used to depict relative gene expression, where blue represents low relative expression and red high relative expression. **C.** Experimental design. PBMCs from healthy donors were treated with panobinostat, romidepsin (FK228) or SIS17 (HDAC11 inhibitor) for 2h, and then treated with TGF-β for 24h prior to stimulation with IFN-β (24h). pSMAD2/3, pSTAT-1, and IRF-1 were evaluated by flow cytometry 24h later. **D-F.** Bar plots with individual data points showing the induction or suppression of IRF1 under various TGF-β, panobinostat, romidepsin (FK228) or SIS17 (HDAC11 inhibitor), and IFN-β, stimulation conditions. * p < 0.05, ** p < 0.005, *** p < 0.0005, **** p < 0.0001, One-way ANOVA, Tukey post-test. IFN: interferon; ISGs: IFN-stimulated genes; LIMMA: linear models for microarray and RNA-Seq data; PBMCs: peripheral blood mononuclear cells.

As TGF-β triggers the expression of HDACs, we investigated their role in modulating the antiviral pathways downstream of IFNs. We analyzed bulk transcriptomic data from a clinical trial of PWH on ART who received the HDAC inhibitor (HDACi) vorinostat^35^, which acts on class I, II and IV histone deacetylases. Induction of IFN signaling, including receptors (IFNAR2, IFNGR1), upstream modulators (STAT1, STAT3), and downstream effector molecules (ISGs with antiviral activity: IRF7, MX1/2, APOBEC3F – Fig 3A), was observed as early as 2 hours post-vorinostat treatment (before its impact on virus reactivation in the periphery) and persisted up to the seventh dose of vorinostat (day 7 + 2h). Of note, regression analysis revealed that expression levels of ISGs 2 hours post-vorinostat administration were inversely correlated with CA-vRNA levels in study participants at the end of follow up *i.e.*, 84 days post initiation of vorinostat treatment (Fig. 3B).

We next validated experimentally the impact of changes on the epigenetic landscape mediated by the HDACi panobinostat (100 nM), SAHA (10 nM) (both pan-HDACi), romidepsin (10 nM) (a potent HDAC1 and HDAC2 inhibitor), and SIS17 (100 nM) (a selective HDAC11 inhibitor), in reversing the impact of TGF-β on the suppression of innate IFN-induced antiviral pathways (Fig 3C). TGF-β significantly induced the phosphorylation of pSMAD2/3 and IFN-β, and IFN-γ significantly induced the phosphorylation of pSTAT1 in CD4^+^ T cells, as compared to untreated conditions (**Extended Data Fig. 3B**). In addition, IFN-β induced significant upregulation of the per cell level (MFI) expression of IRF1 24h post-stimulation, and this was suppressed in cells pre-treated with TGF-β (Fig. 3D–F). However, when each of the aforementioned HDACi were added along with TGF-β and cells were then stimulated with IFN-β, higher expression of IRF1 was detected, similar to IFN-β alone (Fig. 3D–F and **Extended Data Fig. 3C–E**). Similar data were obtained following IFN-γ stimulation in combination with panobinostat (**Extended Data Fig. 3F**). These results support our hypothesis that HDAC inhibition can overcome the impact of TGF-β on IFN-signaling enabling the induction of antiviral pathways.

### Higher gene expression of IRF/STAT1 targets was observed pre-ATI in CA-vDNA^lo^ RMs.

**Fig 4.**
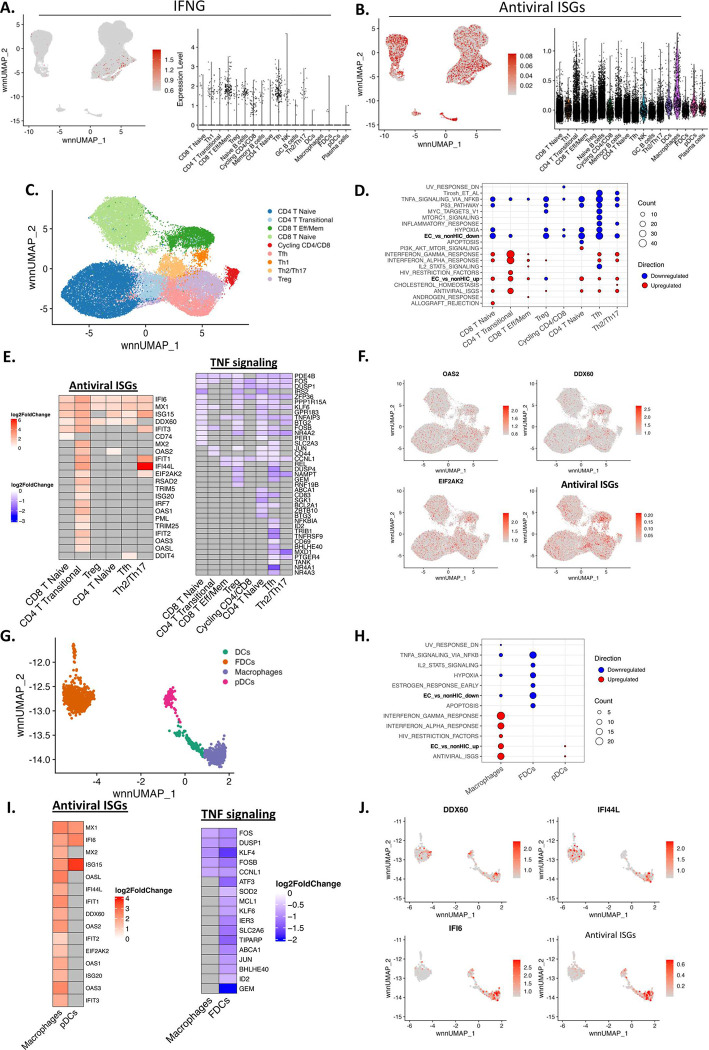
Upregulation of IFNs and antiviral ISGs and downregulation of inflammatory pathways in innate and adaptative cells isolated from LNMCs pre-ATI is associated with lower levels of CA-vDNA post-ATI. **A-B.** UMAP showing the normalized per cell expression of IFN-γ and antiviral ISGs module scores in lymphoid and myeloid cells sourced from LNMCs, respectively. The antiviral ISG module was calculated using the AddModuleScore function from Seurat and a list of 50 antivirals ISGs sourced from Schoggins JW, Rice CM, Curr Opin Virol 2011^61^. **C.** UMAP plot with CD4^+^ and CD8^+^ T cell subsets. CD4^+^ and CD8^+^ T cells from LNMCs were re-clustered and manually annotated based on expression of marker genes. **D.** Overrepresentation analysis of DEGs in T cell subsets between CA-vDNA^lo^ and CA-vDNA^hi^ RMs. Red dots represent pathways upregulated while blue dots represent pathways that are downregulated in CA-vDNA^lo^ RMs. The size of the dots reflects the number of DEGs in each pathway. « EC_vs_nonHIC » pathways include differentially expressed genes from CD4^+^ and CD8^+^ T cells and monocytes of ECs obtained from dos Santos *et al.* (personal communication). Tirosh *et al*., pathway is composed of genes from Tirosh *et al*, 2016^62^. **E.** Heatmap showing the fold change in levels of expression of DEGs belonging to antiviral pathways (left) and TNF-α signaling pathways (right) in selected CD4^+^ and CD8^+^ T cell subsets upregulated (red) or downregulated (blue) in CA-vDNA^lo^ RMs. Genes that did not reach the significance threshold (FDR adjusted p value < 0.1) are represented by grey boxes. **F.** UMAP plot illustrating the expression of the upregulated (red) antiviral ISGs OAS2, DDX60, EIF2AK2, and the overall antiviral ISG module in T cell subsets as described in B. **G.** UMAP plot depicting the clustering of myeloid cells and DCs sourced from LNMCs obtained from combo-treated RMs pre-ATI, including DCs, FDCs, macrophages, and pDCs. Manual annotation of the clusters was performed based on expression of marker genes and compared to a reference-based annotation with the Azimuth package. **H.** Overrepresentation analysis of DEGs in myeloid cell subsets and DCs between CA-vDNA^lo^ and CA-vDNA^hi^ RMs. Red dots represent pathways upregulated while blue dots represent pathways that are downregulated in combo-treated CA-vDNA^lo^ RMs. The size of the dots reflects the number of DEGs in each pathway. **I.** Heatmap showing the fold change in expression of DEGs from antiviral pathways (left) and TNF-α signaling pathways (right) in subsets of myeloid cells and DCs between combo-treated CA-vDNA^lo^ and CA-vDNA^hi^ RMs. **J.** Gene expression of the upregulated (red) antiviral ISGs DDX60, IFI44L, IFI6 and the overall antiviral ISG module in myeloid cells and DCs. The antiviral ISG module was assembled as described under B. ATI: analytical treatment interruption; CA-vDNA: cell-associated viral DNA; CA-vRNA: cell-associated viral RNA; DCs: dendritic cells; DEGs: differentially expressed genes; ECs: elite controllers; FDCs: follicular dendritic cells; FDR: false discovery rate; IFN: interferon; ISG: IFN-stimulated genes; LNMCs: lymph node mononuclear cells; NES: normalized enrichment scores; PBMCs: peripheral blood mononuclear cells; pDCs: plasmacytoid dendritic cells; UMAP: uniform manifold approximation and projection.

Trained immunity involves epigenetic reprogramming of cells of the innate immune system and results in long-term alterations of the effector functions of innate immune cells that impact their response to a subsequent challenge^36,37^. In our study we see that prior exposure to SIV in RMs that received the combo, provided the poised environment to control VL and decay of reservoir (in a subset of RMs), which is parallels mechanisms of trained immunity^36,37^. We used the MultiOme platform that combines single-cell RNA-Seq and ATAC-Seq (**Extended Data Fig. 4A**) to 1) identify cells that triggered the IFN antiviral response leading to decay of viral DNA, and 2) determine if transcriptional differences observed in response to viral rebound post-ATI were associated with changes in chromatin accessibility of the loci that control innate antiviral immunity, a mechanism well-known to promote trained immunity^38^. Clusters of myeloid and lymphoid cell subsets in LNMCs were identified by integrating scRNA-Seq and scATAC-Seq data (**Extended Data Fig. 4B–C**). Semi-automatic cell type annotation was performed by comparing expression of marker genes in each cluster to automatic annotation from SingleR and Azimuth^39,40^ (**Extended Data Fig. 4D–E**). Expression of antiviral genes was observed across all innate (monocytes, DCs, pDCs and FDCs) and adaptive (naïve, memory and effector CD4^+^ and CD8^+^ T cells, B cells) immune subsets (**Extended Data Fig. 4F – Supplementary Table 07**). Of note, while only a small fraction (<2%) of CD4^+^ and CD8^+^ effector T cells expressed mRNA for IFN-γ (Fig. 4A), significantly higher frequencies of cells (>47%) expressed target genes of IFN-γ/STAT1 signaling, including CD4^+^ T cells and myeloid cells (Fig. 4B), which are highly susceptible to HIV/SIV infection. The spreading of IFN-γ signaling to other cell subsets in LNMCs was confirmed by using cell-cell communication analysis^41^, a bioinformatic tool that enables the integration of scRNA-Seq gene expression data to associate the source of the signal with cells that receive that signal, indicative of potential ligand/receptor interactions. We identified Th1, effector CD8^+^ T cells, cycling CD4^+^ and CD8^+^ T cells and Tfh cells in LNMCs as senders of IFN-γ, while the receivers of IFN-γ signaling included all CD4, CD8 and B cell subsets and to a lesser extent macrophages, FDCs and pDCs (**Extended Data Fig. 4G**). Expression of type-I IFN genes was not detected in LNMCs, consistent with reduced chromatin accessibility of these genes as compared to the IFN-γ locus (**Extended Data Fig. 4H**).

We subclustered T cells (Fig. 4C) and myeloid cells (Fig. 4G) and compared genes and pathways that discriminated CA-vDNA^lo^ from CA-vDNA^hi^ RMs. Pathways involving the IFN-γ response, IFN-α, HIV RFs, antiviral ISGs, antigen presentation (CIITA), cell migration (chemokines and integrins), cell cycle, T cell activation/signaling (Fig. 4D), C/EBPβ target genes (*i.e.*, IL-6; **Extended Data Fig. 5A**), and IRF9, a TF upstream of IFN responses, were significantly upregulated in most T cell subsets (naïve CD4^+^ and CD8^+^ T cells, Tregs, Tfh – Fig. 4D) and myeloid cells (macrophages, pDCs - Fig. 4H) from CA-vDNA^lo^ RMs (**Supplementary Table 08**). C/EBPβ is downstream of type-II IFN and triggers IL-6, which in turn induces IRF9 resulting in the amplification of the IFN responses^42^. Representative DEGs of the ISG antiviral pathways for both lymphoid and myeloid cell lineages are shown (OAS2, DDX60, EIF2AK2 - Fig. 4E–F and I–J, respectively). These results suggest that a low frequency of cells producing IFN-γ triggers the molecular machinery involved in conferring a poised innate antiviral state.

On the other hand, T cells and myeloid cells from CA-vDNA^hi^ RMs were enriched in TNF-induced pathways via NF-κB signaling (Fig. 4D–E, H–I – blue plots), as well as in genes of the inflammatory response (TNF, FOS, JUN, DUSP1, and REL) (**Extended Data Fig. 5B–C**). Of note, both CA-vDNA^lo^ and CA-vDNA^hi^ RMs expressed pathways and effector molecules (GZMB, PRF1, TBX21) of the cell-mediated immune response (ZAP70, Fyn) when VL was at undetectable levels. These effector molecules were associated with IFN-γ and were negatively correlated with CA-vRNA levels (**Extended Data Fig. 5D–F**). Hence, while a network of effector molecules of the adaptive immune response was expressed by both groups of RMs is correlated with the control of levels of CA-vRNA in LMNCs, upregulated levels expression of innate antiviral genes was associated with the observed decay in CA-vDNA only in CA-vDNA^lo^ RMs.

### Higher chromatin accessibility for IRF/STAT1 binding sites was observed pre-ATI in CA-vDNA^lo^ RMs.

**Fig 5.**
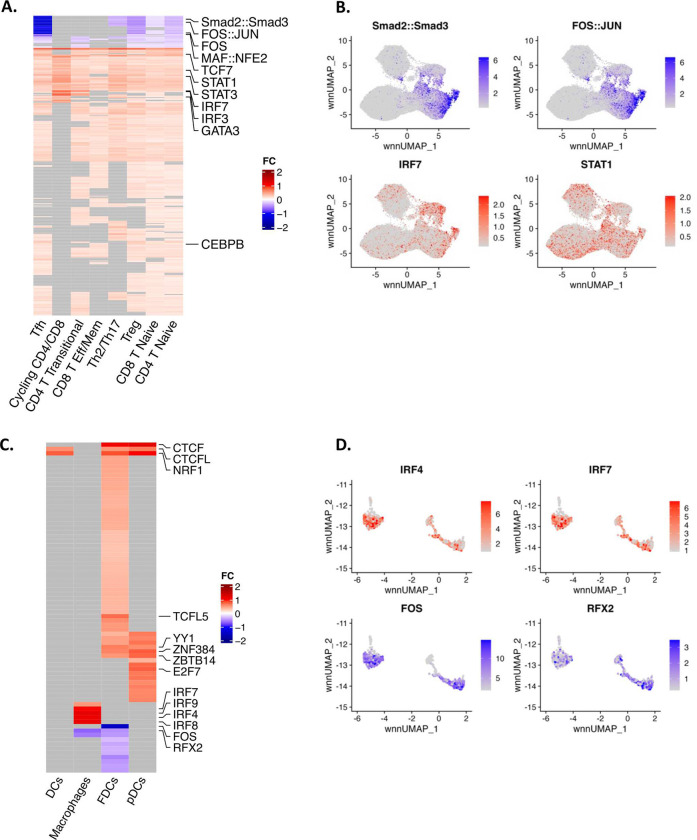
Greater chromatin accessibility of IFN-associated transcription factors (STAT/IRF) binding sites and closed chromatin state of AP-1 family transcription factors binding sites pre-ATI is associated with lower CA-vDNA levels post-ATI. **A.** Average difference of TFs motif chromatin accessibility, *i.e.,* motif activity scores, in CD4^+^ and CD8^+^ T cell subsets from LNMCs isolated from CA-vDNA^lo^ RMs. TFs from the STAT, IRF and AP-1 families are indicated by arrows. Per-cell TFs motif activity was computed with ChromVar. Differentially active motifs were identified by using the Wilcoxon rank sum test on the average differences of scaled TFs motif activity by cell subset and group, with an adjusted p value (FDR) < 0.05. Only the TFs that showed statistical significance in at least two T cell subsets are shown. **B.** UMAP representation of chromatin accessibility of motif binding sites for SMAD2/3 and FOS/JUN and IRF7 and STAT1 in T cells subsets from CA-vDNA^lo^ RMs pre-ATI. Lower levels of chromatin accessibility (*i.e.*, SMAD2, FOS/JUN) are in blue and higher levels of accessibility (*i.e.*, IRF7, STAT1) are in red. **C.** Average differences in TFs activity scores in myeloid cells and FDCs from LNMCs from CA-vDNA^lo^ RMs pre-ATI. TFs motif activity was calculated and compared between groups as described for the T cell subsets (panel A). The top 5 TFs per subset based on highest absolute fold-change are indicated by arrows. **D.** UMAP representation of chromatin accessibility of motif-binding sites for IRF4, IRF7, FIS and RFX2 in myeloid cells and FDCs from combo-treated CA-vDNA^lo^ RMs pre-ATI. Lower levels of chromatin accessibility are in blue and higher levels of accessibility are in red. ATI: analytical treatment interruption; CA-vDNA: cell-associated viral DNA; FDC: follicular dendritic cells; FDR: false discovery rate; LNMCs: lymph node mononuclear cells; RMs: rhesus macaques; UMAP: uniform manifold approximation and projection; TFs: transcription factors.

To identify if the chromatin of the aforementioned TFs was accessible pre-ATI, scATAC-Seq of LNMCs was analyzed. We observed that the enhanced transcriptional activity of IFN loci was concomitant with increased chromatin accessibility of TFs STAT1, IRF1 and IRF7 and their target genes (**Extended Data Fig. 6A, B**) in T cells (Tfh, cycling, effector CD4^+^ and CD8^+^ T cells) from CA-vDNA^lo^ RMs (Fig. 5A–B). Importantly, chromatin accessibility of IRF9, a TF that triggers the expression of other TFs and their target genes with antiviral activity, which is part of the broad antiviral machinery, was significantly higher in CA-vDNA^lo^ RMs. In addition, LTBP1, another C/EBPβ target gene known to maintain TGF-β in its latent form, was upregulated in CA-vDNA^lo^ RMs, while FOS and HDAC9, downstream of active TGF-β signaling, were upregulated in CA-vDNA^hi^ RMs (**Extended Data Fig. 6C–D**).

T cell subsets from CA-vDNA^lo^ RMs presented with decreased chromatin accessibility for the AP-1 family (Fos/Jun, MAF) and SMAD2/3 TF binding sites pre-ATI (**Extended Data Fig. 6E–F**). The comparative accessibility of motif binding sites for these TFs (SMAD, Fos/Jun, IRF7 and STAT1) in T cells is shown in Fig. 5A–B. In the myeloid compartment, the binding sites for IFN-associated TFs (IRF4, 7, 8 and 9) were accessible in macrophages in LNMCs from CA-vDNA^lo^ RMs (Fig. 5C–D). In contrast, chromatin accessibility for IRF7 and STAT1 was observed mostly in T cells with low chromatin accessibility for the SMAD2-SMAD3/AP-1 complex. These data highlight the reciprocal interplay between the IFNs and TGF-β in modulating the activity of the TFs (*i.e.*, AP-1, IRFs, C/EBPβ, SMADs) and the chromatin accessibility of their target genes. This results in the differential modulation of antiviral *versus* TGF-β pathways in CA-vDNA^lo^ and CA-vDNA^hi^ RMs, respectively.

### Protective signatures found in CA-vDNA^lo^ RMs are present in ECs.

**Fig. 6.**
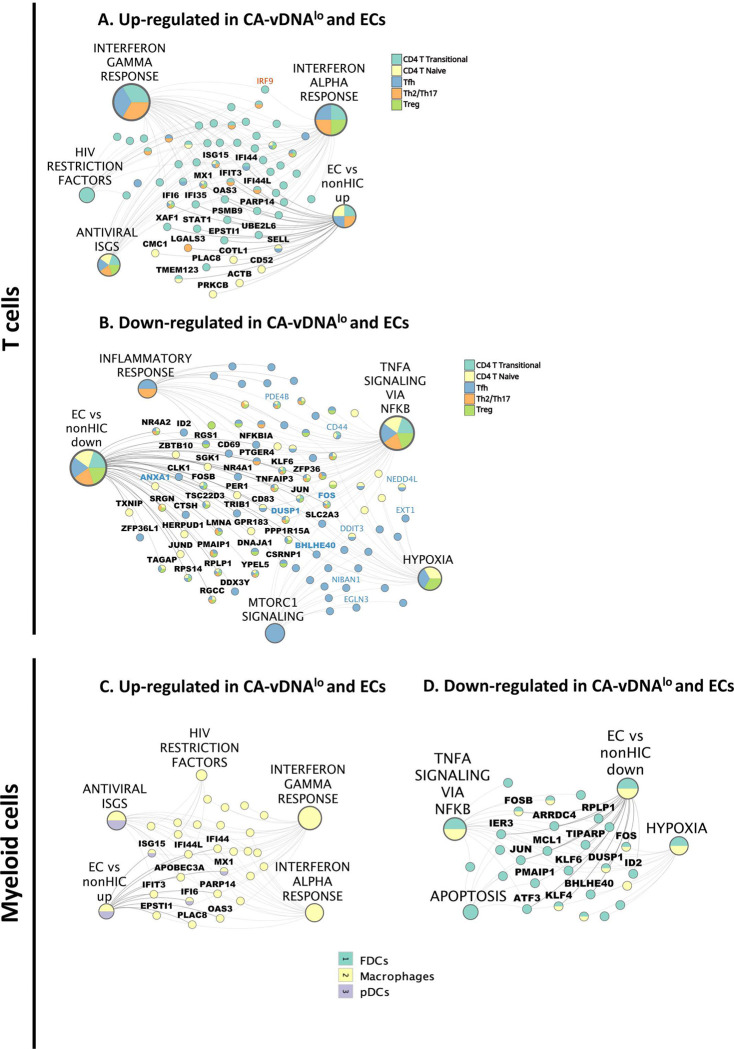
Overlap of gene expression signatures between HIV elite controllers and CA-vDNA^lo^ RMs. **A.** Upregulated pathways in T cell subsets from CA-vDNA^lo^ RMs and ECs. Larger nodes represent pathways, and the size of the nodes reflects the number of DEGs in each pathway. Shared leading edge genes are also represented (small nodes) and bold labeled nodes indicate genes from ECs that overlap with the DEGs from the CA-vDNA^lo^ RMs. Targets of C/EBPβ are labeled in red. Colors inside the nodes indicate the cell subset in which the genes or pathways in question are statistically significant. **B.** Downregulated pathways in T cell subsets from CA-vDNA^lo^ RMs and ECs. Targets of C/EBPβ are labeled in blue. **C.** Upregulated pathways in myeloid subsets and FDCs from CA-vDNA^lo^ RMs and ECs. **D.** Downregulated pathways in myeloid subsets and FDCs from CA-vDNA^lo^ RMs and ECs. CA-vDNA: cell-associated viral DNA; DEGs: differentially expressed genes; ECs: elite controllers; FDC: follicular dendritic cells; RMs: rhesus macaques.

To demonstrate that these signatures of VL control observed in RMs with CA-vDNA^lo^ phenotype that sustained virologic control post-ATI could be extended to PWH who control HIV VL in the absence of ART (Elite controllers: ECs, <75 copies RNA/mL, n = 6), we extracted gene signatures from scRNA-Seq data that were elevated in peripheral blood CD4^+^ T cells, CD8^+^ T cells, and monocytes from ECs when compared to non-HIV controllers (non-HIC, n = 30) from the report of dos Santos *et al.* (dos Santos et al., personal communication). We then assessed if these signatures were observed in T cells (Fig. 4D) and myeloid cells (Fig. 4H) from LNMCs of CA-vDNA^lo^ and CA-vDNA^hi^ RMs. A proportion of genes (40.6%) that were overexpressed in ECs were also upregulated in CA-vDNA^lo^ RMs. These genes included antiviral ISGs, HIV RFs, the IFN-α response, and the IFN-γ response (*e.g.*, ISG15, MX1, IFIT3, and IFI6), all associated with long-term control of plasma VL and low levels of CA-vDNA in LMNCs from combo-treated RMs (Fig. 6A, **Supplementary Table 09**). This overlap was also observed in the myeloid compartment (Fig. 6C, **Supplementary Table 09**). Similar to what we observed in CA-vDNA^lo^ RMs, several C/EBPβ target genes were upregulated in T cells from ECs. They included heightened levels of the target genes of IRF9 and as well the downregulation of the expression of Fos and Jun that were uniquely observed in T cells from ECs. Conversely, downregulated gene signatures in ECs *versus* non-HIV controllers (« EC vs non-controllers down ») overlapped (48.5%) with signatures found in T cells and myeloid cells from LNMCs of CA-vDNA^hi^ RMs. They predominantly included target genes of the TNF signaling cascade via NF-κB and mTORC1, two proinflammatory pathways, AP-1 targets, and TGF-β/SMAD related genes (ANXA1, BHLHE40, JUN, KLF6, LMNA, TSC22D3, ZFP36, ZFP36L1) (Fig. 6 B, 6D). Overall, our findings highlight the interplay between TGF-β and IFN target genes and the relevance of the suppression of TGF-β signaling for the proper generation of IFN-induced antiviral signatures, which can protect CD4^+^ T cells from virus re-seeding following ATI (**Extended Data Fig. 7**).

## Discussion

We previously reported the adaptive immune-mediated mechanisms associated with the long-lasting control of SIV viral rebound post-ATI in RMs that underwent antibodymediated blockade of IL-10 and PD-1 signaling^28^. Herein, we dissected the mechanisms associated with the decay in the size of the SIV reservoir (cell-associated viral DNA) observed in 40% of these RMs, which is key to inform HIV cure strategies. Our findings suggest that the triggering and amplification of the antiviral pathways by IFN-γ and C/EBPβ/IL-6, respectively, conferred protection from infection of bystander CD4^+^ T cells upon ATI, resulting in the CA-vDNA^lo^ phenotype. In contrast, higher plasma levels of TGF-β and the upregulation of the SMAD-signaling pathway led to the epigenetic reprogramming of cells and the blockade of antiviral signaling pathways, resulting in the CA-vDNA^hi^ phenotype post-ATI.

We describe a molecular cascade triggered by an immune intervention that is associated with the long-term control of VL post-ATI. However, this intervention resulted in two phenotypes in terms of SIV-reservoir decay. In CA-vDNA^lo^ RMs, this molecular cascade was characterized by the coordinated epigenetic reprogramming of gene loci related to IRFs, STATs, TFs and C/EBPβ in myeloid and lymphoid cells pre-ATI, consistent with the development of the antiviral signature of trained immunity^42–45^. Conversely, transcriptional activity of the SMAD/AP-1 TFs antagonized chromatin accessibility of IFN loci and expression of ISGs with antiviral activity, leading to maintenance of higher frequencies of cells with vDNA in CA-vDNA^hi^ RMs. The antiviral signature was antigen-independent, as at that time point (pre-ATI), viremia was fully suppressed, and CA-vRNA levels in LNMCs as well as SIV-specific T cell responses were comparable across groups of RMs^28^. Limited frequencies of effector CD4^+^ and CD8^+^ T cells that produced type-II IFN were associated with the induction and persistence of this antiviral signature in most cell subsets found in LNMCs from CA-vDNA^lo^ RMs. Our findings suggest that control of VL and decay of vDNA are regulated by independent mechanisms. While control of VL post-ATI is mostly associated with SIV-specific effector functions^28^, decay of cells with vDNA requires also the upregulation of ISGs, which leads to reduced seeding of CD4^+^ T cells by SIV post-ATI, resulting in lower CA-vDNA levels. We propose that the coordinated activity of C/EBPβ and IL-6 synergizes with ISGs to maintain the CA-vDNA^lo^ phenotype.

### The coordinate transcriptional activity of IRFs and C/EBPβ is required for the control of VL and SIV DNA decay.

Combo treatment was the only study arm that showed the transcriptional upregulation of IRF7 and STAT1, two TFs that regulate IFN production and signaling, respectively, particularly in CD4+ T cells and myeloid cells^21^. The fact that the IFN response and production is restored only in combo-treated RMs suggests that addition of aPD-1 synergized with aIL-10 to trigger transcription of ISGs and increased the frequencies of monocytes and T cells that expressed ISGs, resulting in lower levels of SIV RNA and DNA in CA-vDNA^lo^ RMs. Importantly, this antiviral signature and diminished viral dissemination coincided with heightened chromatin accessibility of IRF/STAT target genes. In addition, our results suggest the role of C/EBPβ in promoting the innate antiviral immune response observed in CA-vDNA^lo^ RMs. C/EBPβ is triggered by type-II IFN^42^ and in turn activates IL-6, a cytokine that was only upregulated in CA-vDNA^lo^ RMs. In addition, IRF9, another C/EBPβ target gene, is an upstream regulator of type-I and -II IFN responses^31^. Heterodimerization of C/EBPβ with AP-1 leads to the inhibition of the pro-inflammatory response triggered by AP-1^46^. Although we do not have direct evidence that C/EBPβ and AP-1 heterodimerize in our experiments, concomitant expression of C/EBPβ and AP-1 was associated to inhibition of expression of AP-1 target genes^46^, similar to our findings in CA-vDNA^lo^ RMs. This activity of C/EBPβ and IL-6 provide a mechanism whereby a few cells LNMCs (2%) that produced type-II IFN could induce expression of ISGs in many (>47%) LNMCs. This synergy between IFN and IL-6 most likely represents a key mechanism that leads to the protection against viral dissemination post-ATI that was observed in CA-vDNA^lo^ RMs.

### TGF-β blunts the IFN response.

CA-vDNA^hi^ RMs presented with elevated TGF-β plasma levels resulting in higher chromatin accessibility of SMAD2/3 target genes pre-ATI. Network analysis of scRNA-Seq and scATAC-Seq data showed that molecular pathways that modulate chromatin structure (SMARCA4), accessibility (HDACs, BRD4), methylation (KDMs) and acetylation (HDACs) of histones, are associated with TGF-β signaling and SMAD2/SMAD3 activity. Specifically, TGF-β signaling upregulates HDACs, including HDAC11, which suppresses the production of type-I IFNs^47^, resulting in the maintenance of latent HIV reservoirs. Our *ex vivo* and *in vitro* results show that addition of an HDACi augments IFN signaling and transcription of antiviral ISGs. These data support a model whereby expression of TGF-β triggers HDACs that inhibit expression of ISGs and thereby prevent the development of innate antiviral effector functions.

### Downregulation of AP-1 and NF-κB and upregulation of antiviral trained immunity in CA-vDNA^lo^ RMs.

Our findings underscore a) the importance of IFNs and expression of ISGs by cells that are the main targets of HIV/SIV infection; and b) the importance of downregulation of AP-1 and NF-κB in immune cells from CA-vDNA^lo^ RMs. IL-1 and TNF are well-known drivers of HIV replication, hence their downregulation in CA-vDNA^lo^ RMs provides a mechanism that can lead to decreased levels of viral seeding post-ATI. In CA-vDNA^hi^ RMs, heightened levels of TGF-β resulting from the expression of SMAD2/3 and AP-1 inhibit IRFs and STAT1 transcription and thereby expression of antiviral ISGs. We observed coordinate transcriptional regulation of genes commonly targeted by SMAD and AP-1 complexes and showed that CA-vDNA^hi^ RMs exhibited heightened activity of proinflammatory pathways, including TNF signaling and hypoxia. AP-1 and NF-κB signaling are critical for the expression of IL-1β and NLRP3, two of the key effector molecules in proinflammatory pathways. TGF-β, which is produced by multiple lineages of white blood cells but predominantly by CD4^+^ Tregs, may provide the balance between these two distinct pathways that are present in the two subgroups of combo-treated RMs. Our findings are generalizable and clinically relevant, as the signatures we observed were echoed in ECs who, similar to the CA-vDNA^lo^ RMs, control viral replication in the long-term and exhibit low frequencies of cells that contain integrated HIV DNA^48^. This imprinting, induced *in vivo* by combo treatment, protected bystander CD4^+^ T cells from infection by rebounding SIV post ATI. Wimmers *et al.*^49^ and Lee *et al.*^50^ showed the importance of epigenetic reprogramming and transcriptional regulation of TF loci that control type-I and type-II IFN production and their role in inducing a state of resistance to SARS-CoV-2 infection *in vivo* and to Zika and Dengue virus infection *in vitro*. Our results and those of Wimmers *et al.*^49^ and Lee *et al.*^50^, highlight significant differences between innate immune pathways that confer resistance to bacterial and fungal infections, *i.e.* AP-1-driven pathways, and those involved in resistance to viral infections, *i.e.,* IRFs and STAT1-driven pathways^37^.

Many immune interventions that have been evaluated for their ability to reduce the HIV reservoir are focused on stimulating T cell-mediated immune responses (e.g., therapeutic vaccines) and/or triggering or mimicking B cell-mediated immune responses (e.g., bNAbs)^51^. Herein we showed the critical role of a novel trained innate immunity antiviral program in shaping immune responses that can inhibit viral replication and dissemination and stimulate the development of long-lasting and effective adaptive immune responses. CA-vDNA^hi^ and CA-vDNA^lo^ RMs received the same intervention and were similarly capable of developing adaptive effector functions to control VL rebound post-ATI. However, trained immunity signatures discriminated between their capacity to modulate SIV reservoir decay, and TGF-β plays a key role in modulating these signatures. The negative impact of TGF-β on radiation cancer immunotherapy in mouse models and on anti-PD-1 immunotherapy in cancer patients has been reported previously^52,53^. The mechanisms underlying this TGF-β mediated inhibition on immune interventions have not been fully defined, but TGF-β has been shown to suppress type 1 and 2 IFN signaling, as well as adaptive immune responses^54–56^, and TGF-β inhibitors in combination with PD-1-blockade are in clinical trials of cancer patients. Moreover, a TGF-β receptor inhibitor was reported to have an impact on SIV latency reactivation in some SIV-infected ART treated RMs although again the mechanisms mediating this reactivation were not defined^14^. Our studies provide a strong rationale and a clear path towards clinical interventions that combine PD-1 and HDAC blockade to restore immune responses that will achieve decay of the HIV reservoir.

Mouse models and clinical trials showed that HDACi and aPD-1 can synergize to clear tumors^57^. Wen *et al.* showed that aPD-1 and chidamide (an HDACi) enhance chemokine expression by T cells and augment the IFN-γ response in immunocompetent preclinical models of NK-T cell lymphoma^58^. Wang *et al.* suggested that the combination of an aPD-1 antibody, an HDACi and anti-VEGF antibodies promoted progression-free survival for 18 weeks in 43% of patients with unresectable, advanced or metastatic colorectal cancer^59^. Based on results in the field of cancer immunotherapy and our own experimental results, we propose that interventions, including HDACi, that reverse immune dysfunction, stimulating i) antigen specific immune responses^28^, ii) the production of IFNs pre-ATI, and iii) inhibit the production and downstream functions of TGF-β, can intercept viral rebound and promote viral reservoir decay post-ATI. These strategies should be considered and explored as part of HIV cure strategies. Our findings set a benchmark and a path for clinical interventions that could help achieve a cure for HIV infection.

Study limitations. Results presented in this study identify novel and previously undescribed mechanisms downstream of the dual blockade of IL-10 and PD-1 that led to long-term viremia control post-ATI in 90% of the treated RMs and decay of the SIV reservoir in 40% of these RMs. This intervention leads to virological, immunological and molecular endpoints similar to those observed in post-treatment controllers and HIV elite controllers, raising the likelihood that this intervention can be used as a therapeutic strategy for HIV Cure. Despite the success of such intervention, the actual molecules cannot be promptly translated to human clinical trials due to the side effects observed in RMs treated with this intervention^28^. However, the data highlighted herein dissects the mechanisms of action of such intervention and identifies novel target molecules and pathways that are readily available and translatable to human clinical trials with minimal side effects. PWH on ART would benefit from these interventions by enhancing both their innate and adaptive immune systems, enabling them to combat not only HIV but also other pathogens.

## Methods

### Materials availability.

This study used novel deimmunized aIL-10 (JES3.12G8) and aPD-1 mAbs (1B8 LC3/HC1), which are proprietary reagents developed by Merck & Co., Inc. (Rahway, NJ, USA). Both aIL-10 and aPD-1 mAbs were the subject of material transfer agreements restrictions as these biologics are currently under investigation in human clinical trials.

### Study approval.

This study was approved by the Emory University Institutional Animal Care and Use Committee (IACUC) via permit 201800047. Experiments were conducted following guidelines set forth by the NIH and Animal Welfare Act in regard to the housing and welfare of laboratory RMs. All possible efforts were taken to minimize the pain and discomfort experienced by RMs.

### Experimental model.

Twenty-eight Indian-origin, specific-pathogen-free (SPF) RMs (25 males, 3 females) were sourced from the Emory National Primate Research Center (ENPRC) colony and single-housed in an animal BSL-2 facility at ENPRC, as previously described^1^. RMs were between 38–50 months old at the time of infection and *Mamu*-B*07^−^ and Mamu-B*17^−^. Some RMs were *Mamu*-A*01^+^. RMs were infected, treated and followed up for virological and immunological outcomes, as previously described^28^. The analysis presented herein was focused on immune responses and virological readouts in plasma, PBMCs and LNMCs pre-ATI and at 24 weeks post-ATI. Sample collection and processing, formulation of the aIL-10 and aPD-1 mAbs, pharmacokinetics and pharmacodynamics (PK/PD) of aPD-1 and aIL-10 mAbs, levels of anti-drug antibodies (ADA), plasma IL-10 levels, aPD-1 receptor occupancy, complete blood counts (CBC), blood chemistry, SIV VL, measurement of CA-SIV-vRNA and DNA levels^6^, and measurement of 2-LTR circles were all performed as previously described^28^.

### Downstream analysis strategy.

Immune parameters that were examined (plasma cytokines and CD4^+^ and CD8^+^ T cell features from LNs and PBMCs) were those that were specifically and significantly modulated by combo treatment as compared to control and/or treatment with aIL-10 alone (p < 0.05) at week 0 and 24 weeks post-ATI. These parameters were then correlated with LN CA-vRNA 24 weeks post-ATI, which was used as a surrogate marker of VL control in tissues.

### Bulk RNA-Seq.

100,000 PBMCs or LNMCs from RMs were lysed directly into 700 l of QIAzol reagent. RNA was isolated using the RNeasy Micro Kit (QIAGEN, Hilden, Germany) with on-column DNase digestion. RNA quality was assessed using an Agilent Bioanalyzer and total RNA was used as input for cDNA synthesis using the Clontech SMART-Seq v4 Ultra Low Input RNA kit (Takara Bio, Shiga, Japan) according to the manufacturer’s instructions. Amplified cDNA was fragmented and appended with dual-indexed bar codes using the Nextera XT DNA Library Preparation kit (Illumina, San Diego, CA). Libraries were validated by capillary electrophoresis on an Agilent 4200 Tape Station, pooled at equimolar concentrations, and sequenced on an Illumina NovaSeq6000 at 100SR, yielding 20–25 million reads per sample. The quality of reads was evaluated using Fast QC (https://www.bioinformatics.babraham.ac.uk/projects/fastqc/). Reads were aligned using STAR v2.7.3.^63^. The STAR index was built by combining genome sequences for *Macaca mulatta* (Mmul10 Ensembl release 100). Transcript abundance estimates were calculated internal to the STAR aligner using the htseq-count algorithm^64^. The ReadsPerGene files were used to generate counts in the htseq format using a custom script that also converted the Ensembl ID to gene names using the gtf file. These files were imported in DESeq2 using the DESeqDataSet- FromHTSeqCount function. DESeq2 was used for normalization^65^, producing both a normalized read count table and a regularized log expression table. Regularized log expression values were obtained using the rlog function with the parameters blind = FALSE and filtType = parametric. Thresholds of padj < 0.05, fold-change > 1.5 and lfcSE < 1 were used to identify genes with statistically significant differences in expression levels. The input for GSEA was the regularized log expression values obtained from DESeq2, which was filtered to remove genes with mean expression levels of 0. Regularized log expression values were also used to generate heatmaps using the Complex Heatmap R library^66^. GSEA was performed using a compiled set of pathways from public databases, including MSigDB version 5.1 (http://software.broadinstitute.org/gsea/msigdb/) and blood cell marker signatures. The GSEA Java desktop program was downloaded from the Broad Institute (http://www.broadinstitute.org/gsea/index.jsp) and used with GSEA pre-ranked module parameters (number of permutations = 1,000; enrichment statistic = weighted; seed for permutation = 111; 10 ≤ gene set size ≤ 5,000). We used the Dynet Analyzer application implemented in Cytoscape version 3.6.0 to generate gene interacting networks that highlight overlapping genes between the different enriched modules. Sample-level enrichment analysis ^67^ was used to investigate the enrichment of pathways in individual RMs upon the different interventions. Briefly, the expression of all genes in a specific pathway was averaged across samples and compared to the average expression of 1,000 randomly generated gene sets of the same size. The resulting Z score was then used to reflect the overall perturbation of each pathway in each individual sample. Data were visualized using ggplot2 (version 3.3.2) in RStudio (version 1.4.1103) with custom code.

### Construction of single-cell multiome, ATAC, and gene expression libraries.

PBMCs and LNMCs nuclei were isolated according to the manufacturer’s instructions using Chromium Nuclei Isolation Kit (10x Genomics, Pleasanton, CA). Single-cell multiome ATAC and gene expression (GEX) libraries were prepared using the Chromium Single Cell Multiome ATAC + Gene Expression platform (10X Genomics). 8,000 nuclei were used in each sample. Isolated nuclei were transposed and partitioned into Gel Beads-in-emulsion (GEMs) using the 10x Chromium Controller and Next GEM Chip J. ATAC and GEX libraries were generated from the same pool of pre-amplified transposed DNA/cDNA. Representative traces and quantitation of both libraries were determined using Bioanalyzer High Sensitivity DNA Analysis (Agilent, Santa Clara, CA). ATAC and GEX libraries were sequenced respectively on Illumina NovaSeq S2 and S4 systems, using 10x’s recommended parameters. The 10x Barcodes in each library type were used to associate individual reads back to the individual partitions and thereby to each single nucleus.

### Analysis of single-cell multiome data (scRNA-seq and scATAC-Seq).

Raw reads from 10X Chromium Single Cell Multiome ATAC and Gene Expression libraries were aligned to the reference genome using the Cell Ranger ARC Count v2.0.2 pipeline. The aggregated counts file was then imported into Seurat 5.01. Quality control parameters were applied to define high-quality cells. These parameters included: RNA counts between 500 and 25,000, detected genes > 300, mitochondrial reads < 25%, fragment counts between 1,000 and 70,000, a ratio of mononucleosomal to nucleosome-free fragments < 2, transcription start site (TSS) enrichment score > 2, fragments in peaks > 20%, and reads in genomic blacklist regions < 0.05. Following the removal of low-quality cells, DoubletFinder was used to identify and exclude doublet cells. Peak calling was performed using the CallPeaks function. Data normalization and dimensional reduction were performed independently on the gene expression (RNA) and chromatin accessibility (ATAC) datasets, and then integrated using the weighted-nearest neighbor method (WNN). Clustering was performed on the integrated uniform manifold approximation and projection (UMAP) dimension reduction, and manual annotation was performed using cell type-specific gene markers. CD4^+^ and CD8^+^ T cells were further re-clustered to improve the identification of specific subsets of T cells. DEGs between conditions per cell type were identified using the Wilcoxon rank sum test, a minimum frequency of cells in the clusters = 1%, and an adjusted p value (FDR) < 0.1. A per-cell motif binding site activity score was calculated using chromVAR, utilizing a collection of 746 transcription factors from the JASPAR database^68^. Differences in motif activity per timepoint and group were identified using the FindMarkers function with an adjusted p value (FDR) < 0.05.

### Flow cytometry.

Cryopreserved PBMCs and LNMCs (10^6^ per test) were thawed and stained with anti-rhesus or anti-human mAbs that are known to be cross-reactive with RMs cells and were validated in databases maintained by the Nonhuman Primate Reagent Resource. An antibody panel to evaluate antiviral responses was used to stain PBMCs and LNMCs pre-ATI and included CD95 BV605, BioLegend (San Diego, CA) Cat# 305628, Clone DX2; CD3 BUV395, Becton-Dickinson (BD; Franklin Lakes, NJ) Cat# 564117, Clone SP34–2; Live/Dead BV510, BD Cat# 564406; CD8 BUV496, BD Cat# 612942, Clone RPA-T8; CD28 BUV737, BD Cat# 612815, Clone CD28.2; CD14 BV786, BD Cat# 563698, Clone M5E2; CD16 BUV661, BD Cat# custom 3G8; CD4 PerCP/Cy5.5, BD Cat# 552838, Clone L200; pSTAT3 PE, BD Cat# 558557, Clone 4/P-STAT3; pIRF7 (pS477/pS479) A647, BD Cat# 558630, Clone K47–671; p38 (pT180/pY182) PE-Cy7, BD Cat# 560241, Clone 36/p38; pStat5 PE-CF594, BD Cat# 562501, Clone 47/Stat5(pY694); pIRF3, Abcam (Cambridge, United Kingdom) Cat# ab138449, polyclonal; pStat1 AF488, BD Cat# 612596, Clone K51–856; donkey anti-rabbit BV421, BioLegend Cat# 406410, polyclonal Ig. Cell surface staining was performed at 37°C for 20 min. Staining was stopped by adding 50 μL of cold 1 × TFP Fix/Perm Buffer to the cells (BD Cat# 565575) and mixing gently. Cells were then fixed and permeabilized for 40 min at 2–8°C. Cells were washed and rehydrated twice (500 *g* for 5 min) in 200 μL of PBS + 2% FBS buffer. Fifty μL of ice-cold Perm Buffer III were added, cells were mixed gently and incubated on ice for 15 min. Cells were then washed twice with 200 μL of 1 × TFP Perm/Wash Buffer and all residual buffer was removed. Intracellular staining was performed by adding mAbs to 25 μL of master mix diluted in 1 × TFP Perm/Wash Buffer, followed by gentle mixing. Cells were incubated for 30 min at 2–8°C and washed with 200 μL 1× TFP Perm/Wash Buffer by centrifugation at 500 *g* for 5 min at 2–8°C. Finally, cells were resuspended in 100 μL of PBS 2% fetal bovine serum (FBS). Acquisition was performed on a minimum of 100,000 live cells on a A5 Symphony flow cytometer driven by BD FACSDiva software (BD Biosciences).

### Assays for validation.

**A. IFN signaling protects bystander CD4**^**+**^ **T cells from HIV infection *in vitro.*** Memory CD4^+^ T cells were isolated from healthy human donors (n = 6). Cryopreserved PBMCs were thawed and rested overnight in AIM V medium + 10% Serum Replacement (Corning Life Sciences, Durham, NC) + 10 mM HEPES. Memory CD4^+^ T cells were isolated using the EasySep Human CD4^+^ T Cell Enrichment Kit (STEMCELL Technologies, Vancouver, Canada). One half of memory CD4^+^ T cells was stimulated for up to 16h with 5 ng/mL of IFN-β (Peprotech, Cranbury, NJ) while the other half remained unstimulated. These cells were further divided into 2 pools: unlabeled CD4^+^ T cells and CD4^+^ T cells labeled with CellTrace Violet (CTV). Unlabeled CD4^+^ T cells were infected with HIV-1 (p89.6 dual-tropic strain). After infection, cells were cultured in the presence of saquinavir and mixed with autologous CTV-labeled CD4^+^ T cells in a 1:1 ratio). Staining for HIV-1 p24 was performed on day 4 post-infection using BD Phosflow Fix Buffer I (BD) and BD Phosflow Perm Buffer III (BD) according to the manufacturer’s instructions. The staining panel comprised Live/dead BV510, Life Technologies (Carlsbad, CA) Cat# L34957; CD3 BUV615, BD Cat# 612992, Clone UCHT1; CD4 BV605, BioLegend Cat# 317438, Clone OKT4; CD8 BUV737, BD Cat# 564629, Clone SK1; CD45RA BV650, BioLegend Cat# 304136, Clone HI100; CD27 APC-eFluor 780, eBioscience (San Diego, CA) Cat# 47027942, Clone O323; CCR7 BUV563, BD Cat# 741317, Clone 3D12; MX2 AF488, Santa Cruz Biotechnology (Dallas, TX) Cat# 271527, Clone H-7; IFIT1 APC, Novus Biologicals (Centennial, CO) Cat# NBP2–71005APC, Clone OTI3G8; APOBEC3G AF700, Novus Biologicals Cat# NBP1–77206AF700, polyclonal; pSTAT1 (p701) PE-CF594, BD Cat# AB_2737715, Clone 4a; pIRF3 (pSer396) PerCP, Bioss (Woburn, MA) Cat# bs-3195R-PerCP, polyclonal; pIRF7 (pSer471+pSer472) AF350, Bioss Cat# bs-3196R-A350, polyclonal; HIV-1 core antigen FITC, Beckman Coulter (Brea, CA) Cat# 6604665, Clone KC57, and CTV BV421, Thermo Fisher Scientific (Waltham, MA) Cat# C34571. Cell surface staining was performed at 37°C for 20 min in 25 μL of PBS + 2% FBS buffer. Cells were washed with 150 μL of PBS + 2% FBS and were fixed in 50 μL of Phosflow Fix Buffer I (BD) at 4°C for 30 min. Cells were washed again with 150 μL of PBS + 2% FBS and then permeabilized in 50 μL of Phosflow Perm Buffer III (BD) on ice for 15 min. Cells were washed twice with 150 μL of PBS +2% FBS at 500 *g* for 5 min at room temperature. Intracellular staining was performed by adding the intracellular antibody mix in 25 μL of master mix diluted in 1x permeabilization buffer and mixing gently. Cells were incubated for 60 min at 4°C and washed with 150 μL of 1x permeabilization buffer. Cells were then resuspended in 100 μL of PBS 2% FBS. Acquisition was performed on a minimum of 100,000 live cells on an A5 Symphony flow cytometer driven by FACSDiva. Acquired data was initially analyzed using FlowJo version 10.8.1. **B. TGF-β abrogates IFN-induced antiviral signatures and makes cells susceptible to HIV infection.** Memory CD4^+^ T cells were isolated from healthy human donors (n=6) as described above and were then subjected to the following conditions: a) unstimulated; b) stimulated with 2 ng/mL TGF-β (Peprotech, Cat# 100–21) for 24h; c) stimulated with 0.2 or 2 ng/mL IFN-β; and d) stimulated with 2 ng/mL TGF-β in presence or absence of anti-TGF-β antibody (Merck & Co., Inc., Rahway, NJ, USA) for 24h and then with 0.2 or 2 ng/mL IFN-β for 24h. Stimulated CD4^+^ T cells were then infected with HIV (p89.6 dual tropic strain) by spinoculation in presence of saquinavir. Activation of TGF-β and IFN-β signaling pathways (pSMAD2/3 and pSTAT1, respectively) was assessed at 20 min post stimulation, induction or suppression of ISGs was assessed at 16h post IFN-β and TGF-β stimulation, and HIV p24 levels were assessed 4 days post infection. Cell surface and intracellular staining were performed as above. The staining panel comprised Live/dead BV510, Life Technologies Cat# L34957; CD4 BV605, BioLegend Cat# 317438, Clone OKT4; CD8 BUV737, BD Cat# 564629, Clone SK1; CD45RA BV650, BioLegend Cat# 304136, Clone HI100; CD27 BUV496, BD Cat# 751678, Clone O323; CCR7 BUV563, BD Cat# 741317, Clone 3D12; IFIT1 APC, Novus Biologicals Cat# NBP2–71005APC, Clone OTI3G8; APOBEC3G AF700, Novus Biologicals Cat# NBP1–77206AF700, polyclonal; pSTAT1 (p701) AF488, BD Cat# AB_2737715, Clone 4a; pSMAD2(pS465/pS467)/pSMAD3 (pS423/pS425) PE-CF594, BD Cat# 562697, Clone O72–670; p24 RD1, Beckman Coulter Cat# 6604667, Clone KC57; PD1 BV711, BD Cat# 564017, Clone EH12.1; BCL2 BUV395, Cat# custom, Clone Bcl-2/100. Acquisition and analysis of flow cytometry data were performed as above. **C. TGF-β suppresses IFN-dependent antiviral state through HDACs and promotes HIV infection.** To validate our *ex vivo* findings, PBMCs were isolated from fresh blood from healthy human donors (n=5). Cells were cultured in AIMV + 10% Serum Replacement (Corning Cat# 355500) + 10 mM HEPES overnight. Cells were then subjected to the following conditions: i) unstimulated; or stimulated with HDAC inhibitors (HDACi), such as ii) panabinostat (100 nM), iii) SAHA (10 nM), iv) romidepsin (10 nM), and v) SIS17 (100 nM) individually for 24h only; vi) TGF-β only (2 ng/mL) for 24h; vii) IFN- β (1 ng/mL) only for 24h; viii) IFN-γ (1ng/mL) only for 24h; ix) TGF-β (2 ng/mL) for 24h and then IFN- β (1 ng/mL) for 24h; x) TGF-β (2 ng/mL) for 24h and then IFN-γ (1 ng/mL) for 24h; xi) individual HDACi for 2h, then TGF-β (2 ng/mL) for 24h, and then, IFN- β (1 ng/mL) for 24h; and individual, xi) HDACi for 2h, then TGF- β (2 ng/mL) for 24h, and then, IFN- γ (1 ng/mL) for 24h. HDAC inhibitors and TGF- β were removed after 24h of stimulation, just before the start of IFN-β or IFN- γ stimulation. Activation of the TGF- β and IFN- β signaling pathways (pSMAD2/3 and pSTAT1, respectively) was assessed at 20 min post-stimulation. Induction or suppression of ISGs was assessed at 16h post IFNs and TGF- β stimulation. Staining was done as per BD staining protocol with BD Phosflow Fix Buffer I (BD, Cat# 557870) and BD Phosflow Perm Buffer III (BD, Cat# 558050). The staining panel included Live/dead BV510, Life Technologies Cat# L34957; CD3 BUV805, BD Cat# 612895, Clone UCHT1; CD4 BV605, BioLegend Cat# 317438, Clone OKT4; CD8 BUV737, BD Cat# 564629, Clone SK1; CD45RA BV650, BioLegend Cat# 304136, Clone HI100; CD27 BUV615, BD Cat# 751685, Clone O323; CCR7 PE-CF594, BD Cat# 562381, Clone 2-L1-A; IRF7 AF488, Novus biologicals Cat# NBP306987AF488, Clone 3D9; IFIT1 APC, Novus biologicals Cat# NBP2–71005APC, Clone OTI3G8; pSTAT1 (p701) RB780, BD Cat# 569144, Clone 4a; pSmad2 (pS465/pS467)/Smad3 (pS423/pS425) R718, BD Cat# 567080, Clone O72–670; H3K27Ac Pacific Blue, Cell signaling Cat# 23349, Clone D5E4; IRF1 PE, BD Cat# 566322, Clone 20/IRF-1; PD-1 BV711, BD Biosciences Cat# 564017, Clone EH12.1; HLA-DR BV786, BD Biosciences Cat# 564041, Clone G46–6; CD19 BUV395, BD Biosciences Cat# 563549, Clone SJ25C1; CD14 BV570, BioLegend Cat# 301832, Clone M5E2; CD16 BUV661, BD Biosciences Cat# 741693, Clone B73.1; CD56 PE-Cy5, BD Biosciences Cat# 555517, Clone B159. Surface Staining was performed at 37°C for 20 minutes in 25 μL of PBS + 2% FBS buffer. Cells were washed with 150 μL of PBS + 2% FBS buffer. Cells were fixed in 50 μL of Phosflow Fix Buffer I (BD, Cat# 557870) at 4°C for 30 min. Cells were washed again with 150 μL of PBS + 2% FBS buffer and then permeabilized in 50 μL of cold Phosflow Perm Buffer III (BD, Cat# 558050) on ice for 15 min. Cells were washed twice with 150 μL of 1x permeabilization buffer (Invitrogen Cat# 00–8333), and all residual buffer was removed. All centrifugation steps for staining were performed at 500 × g for 5 min at room temperature. Intracellular Staining was performed by adding the intracellular antibody mix in 25 μL of master mix diluted in 1x permeabilization buffer (Invitrogen Cat# 00–8333) and mixed gently. Cells were incubated for 60 min at 4°C and washed with 150 μL of 1x permeabilization buffer. Finally, cells were resuspended in 100 μL of PBS for acquisition. Acquisition was performed on a minimum of 100,000 live cells on a A5 Symphony (BD Biosciences) driven by BD FACSDiva software.

All flow cytometry data was analyzed using FlowJo version 10.8.1. Data analysis was performed by using manual gating frequencies and MFIs (median fluorescence intensity) are shown or by using UMAP followed by cluster analysis. Representative cytograms are shown for each panel in the respective figures.

### Measurement of cytokines.

Meso Scale MULTI-ARRAY Technology (Meso Scale Discovery, Rockville, MD) was used for evaluation of cytokine levels. A cytokine panel containing the following analytes was screened: CTAK, ITAC, IL-10, IL-16, IL-17A, IL-18, IL-4, IP-10, MCP-1, MCP-2, MIP-3α, GROA, IL-22, IL-7, IL-8, TGF-β1, TGF-β2, TGF-β3, Fractalkine, IFN-α, IFN-γ, IL-15, IL-2, IL-6, IL-9, MIP-1α, and TNF-α using 25 μL of serum from each donor in duplicates and following the manufacturer’s instructions. Samples were randomized to avoid batch effects. Results were extrapolated from standard curves for each analyte and plotted in pg/mL using the DISCOVERY WORKBENCH v4.0 software (Meso Scale Discovery). K-means and Gap statistics^69^ were computed to identify clusters of cytokines that were modulated by combo treatment at different time points. These were then associated with CA-vRNA levels in LNMCs 24 weeks post-ATI.

### Statistical Analysis.

Identification of the features modulated by combo treatment versus controls and/or the aIL-10 group pre-ATI and at 9/24 weeks post-ATI was performed using a linear regression model controlling for group staggering. Features with adjusted p value < 0.05 (Holm–Bonferroni) were considered significantly different. Once combo-specific features were selected, a Spearman correlation test versus LN CA-vRNA levels 24 weeks post-ATI was performed, resulting in the identification of specific features that were positively or negatively associated with this outcome. Features exhibiting correlated associations (FDR < 0.1) were also examined for their correlation with plasma VL (copies/mL) at 24 weeks post-ATI. Area under the curve (AUC) was also measured to compare the longitudinal distribution of features, including VL. Modified Kaplan-Meier curves were constructed based on the sum of RMs that achieved VL < 1,000 copies/mL at least once in each group. Note that the aIL-10 group included 9 RMs instead of 10, as one animal was necropsied earlier. The minimal set of features modulated by combo treatment and associated with CA-vRNA levels 24 weeks post-ATI was identified through feature selection using Least Absolute Shrinkage and Selection Operator (Lasso) regression. The shorthand representation of statistical significance is as follows: *P < 0.05; **P < 0.01; ***P < 0.001; ****P < 0.0001. Data showing average statistical outcomes are represented as mean ± SEM and population sizes are listed within the figure legends for each analysis. Statistical analyses were performed using R 4.2.2 with rstatix package version 0.7.2 and GraphPad Prism 9.4.0 (GraphPad Software, Boston, MA). Plots were generated with the R package ggplot2 or GraphPad Prism. Feature selection was used to reduce the number of input variables with a predictive model for control of viral rebound^20^.

## Data Availability

Raw and processed data files from bulk RNA-seq and scMultiome (scRNA-seq and scATAC-seq) are available at Gene Expression Omnibus (GEO) database. Bulk RNA-seq accession: GSE279313; scMultiome accession: GSE279314.
